# Navigated, Robot-Driven Laser Craniotomy for SEEG Application Using Optical Coherence Tomography in an Animal Model

**DOI:** 10.3389/frobt.2021.695363

**Published:** 2021-06-30

**Authors:** Fabian Winter, Tobias Wilken, Martin Bammerlin, Julia Shawarba, Christian Dorfer, Karl Roessler

**Affiliations:** ^1^ Department of Neurosurgery, Medical University of Vienna, Vienna, Austria; ^2^ Advanced Osteotomy Tools, Basel, Switzerland

**Keywords:** robotic, navigated, laser, craniotomy, SEEG, epilepsy surgery, depth electrodes

## Abstract

**Objectives:** We recently introduced a navigated, robot-driven laser beam craniotomy for use with stereoelectroencephalography (SEEG) applications. This method was intended to substitute the hand-held electric power drill in an *ex vivo* study. The purpose of this *in vivo* non-recovery pilot study was to acquire data for the depth control unit of this laser device, to test the feasibility of cutting bone channels, and to assess dura perforation and possible cortex damage related to cold ablation.

**Methods:** Multiple holes suitable for SEEG bone channels were planned for the superior portion of two pig craniums using surgical planning software and a frameless, navigated technique. The trajectories were planned to avoid cortical blood vessels using magnetic resonance angiography. Each trajectory was converted into a series of circular paths to cut bone channels. The cutting strategy for each hole involved two modes: a remaining bone thickness mode and a cut through mode (CTR). The remaining bone thickness mode is an automatic coarse approach where the cutting depth is measured in real time using optical coherence tomography (OCT). In this mode, a pre-set measurement, in mm, of the remaining bone is left over by automatically comparing the bone thickness from computed tomography with the OCT depth. In the CTR mode, the cut through at lower cutting energies is managed by observing the cutting site with real-time video.

**Results:** Both anesthesia protocols did not show any irregularities. In total, 19 bone channels were cut in both specimens. All channels were executed according to the planned cutting strategy using the frameless navigation of the robot-driven laser device. The dura showed minor damage after one laser beam and severe damage after two and three laser beams. The cortex was not damaged. As soon as the cut through was obtained, we observed that moderate cerebrospinal fluid leakage impeded the cutting efficiency and interfered with the visualization for depth control. The coaxial camera showed a live video feed in which cut through of the bone could be identified in 84%.

**Conclusion:** Inflowing cerebrospinal fluid disturbed OCT signals, and, therefore, the current CTR method could not be reliably applied. Video imaging is a candidate for observing a successful cut through. OCT and video imaging may be used for depth control to implement an updated SEEG bone channel cutting strategy in the future.

## Introduction

In recent years, frameless procedures have been increasingly used as an alternative to frame-based procedures to implant depth electrodes (Murphy et al., 2002; Mehta et al., 2005; Dorfer et al., 2014; Dorfer et al., 2017). Frame-based procedures are accurate and safe but are limited in certain trajectories and can restrict access to the surgical field (Spire et al., 2008; Cardinale et al., 2013; Dorfer et al., 2017).

Building the drill hole is one of the crucial surgical steps for the implantation of depth electrodes for invasive monitoring in epilepsy surgery. An electric power drill is used for this in frameless procedures (Ortler et al., 2011; Roessler et al., 2020). However, small deviations in drilling may cause large deviations in target point accuracy. Robot-guided contact-free laser osteotomy has been recently investigated mainly in the fields of oral and maxillofacial surgery (Stübinger et al., 2009; Baek et al., 2015; Augello et al., 2018a; Berg et al., 2019). Data in neurosurgery is limited.

We recently introduced a navigated robot-driven laser beam in an *ex vivo study,* replacing the role of a hand-held electric power drill (Roessler et al., 2020). The purpose of this *in vivo* pilot study was to acquire data for this device’s depth control unit. This study aimed to identify cut through (CTR) images and to evaluate dura and cortex damage in a live specimen.

## Materials and Methods

This study was approved by the local Department de Territori I Sostenibilitat (10076; FUE-2018-00726444 I ID KX68K1DZV). The surgeon performing the procedure is accredited with educational and training courses in laboratory animal science with the EU Function A certificate.

### Preoperative Planning

**FIGURE 1:**
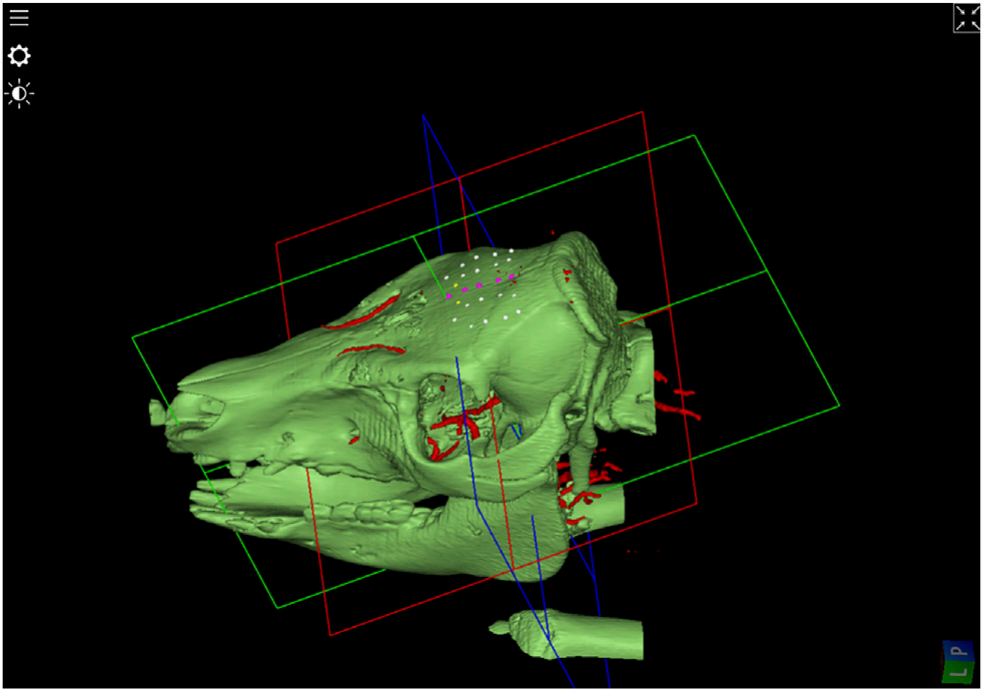
3D-Model of bone surface derived from the CT-image. In addition, after image registration, blood vessels extracted from Angio-MR data were visualized in red. SEEG-trajectory planning in the upper portion of the pig’s skull are shown in white avoiding blood vessels and in purple passing vessels and in yellow under an angle of around 45°.

Two pigs underwent preoperative computed tomography performed with 1 mm axial resolution and magnetic resonance angiogram with 2.2 mm imaging (Figure 1). Using surgical planning software (Suite Version 2.15.2, ImFusion GmBH, Munich, Germany), we planned multiple bone channels on the superior portion of the pig craniums. Trajectories were planned to avoid cortical blood vessels and were planned with a cutting angle < 45 degrees.

### Anesthesia

According to the test facility’s standard of care protocols, the animals were prepared for the operation under general anesthesia, which conforms to the European requirements (Directive EU/2010/63) and the United States Food and Drug Administration Good Laboratory Practice regulations, 21 CFR 58. Pigs were sedated with an intramuscular administration of a combination of dexmedetomidine (0.03 mg/kg), midazolam (0.3 mg/kg), and butorphanol (0.3 mg/kg). Anesthesia was induced intravenously with propofol (1 mg/kg), and pigs had tracheal intubation and mechanical ventilation. Isoflurane 2–4% was used to maintain anesthesia throughout the study.

### Registration Procedure

**FIGURE 2:**
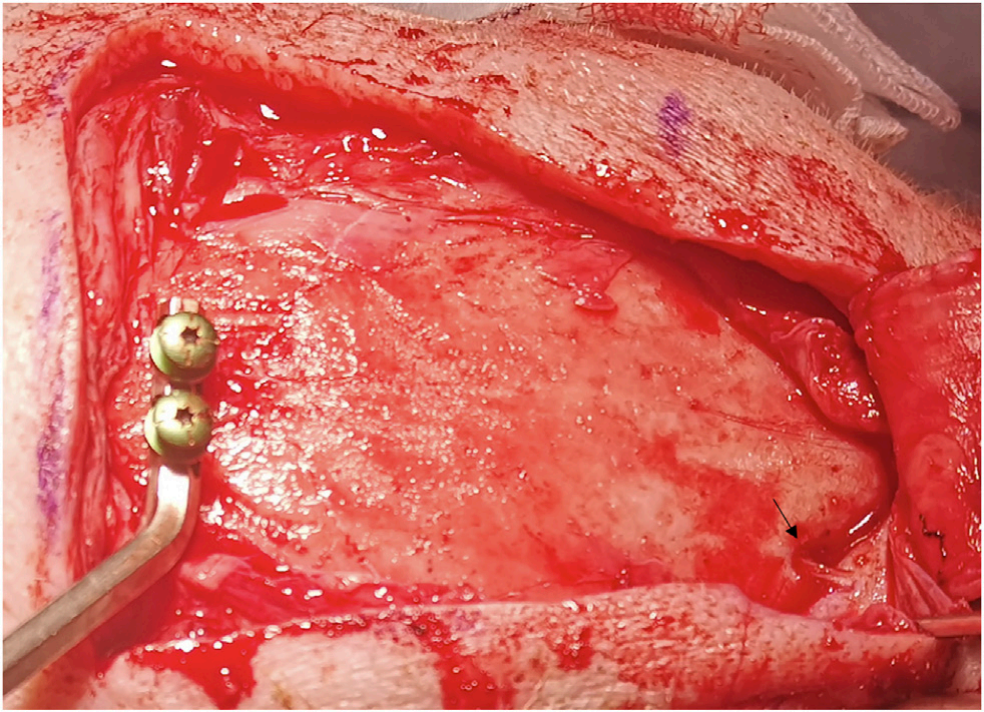
Exposing the supraorbital nerve (arrow) for registration landmarks.

Both pigs were placed in a prone position and prepared with endotracheal intubation. After the cranium exposure at the region of interest for each pig, a patient marker was placed on the pig skull’s apex. The supraorbital nerve was exposed and used for a registration landmark (Figure 2). Registration to the navigational system with mean root square precisions below 1 mm was performed.

### Frameless Laser Craniotomy Tool

**FIGURE 3:**
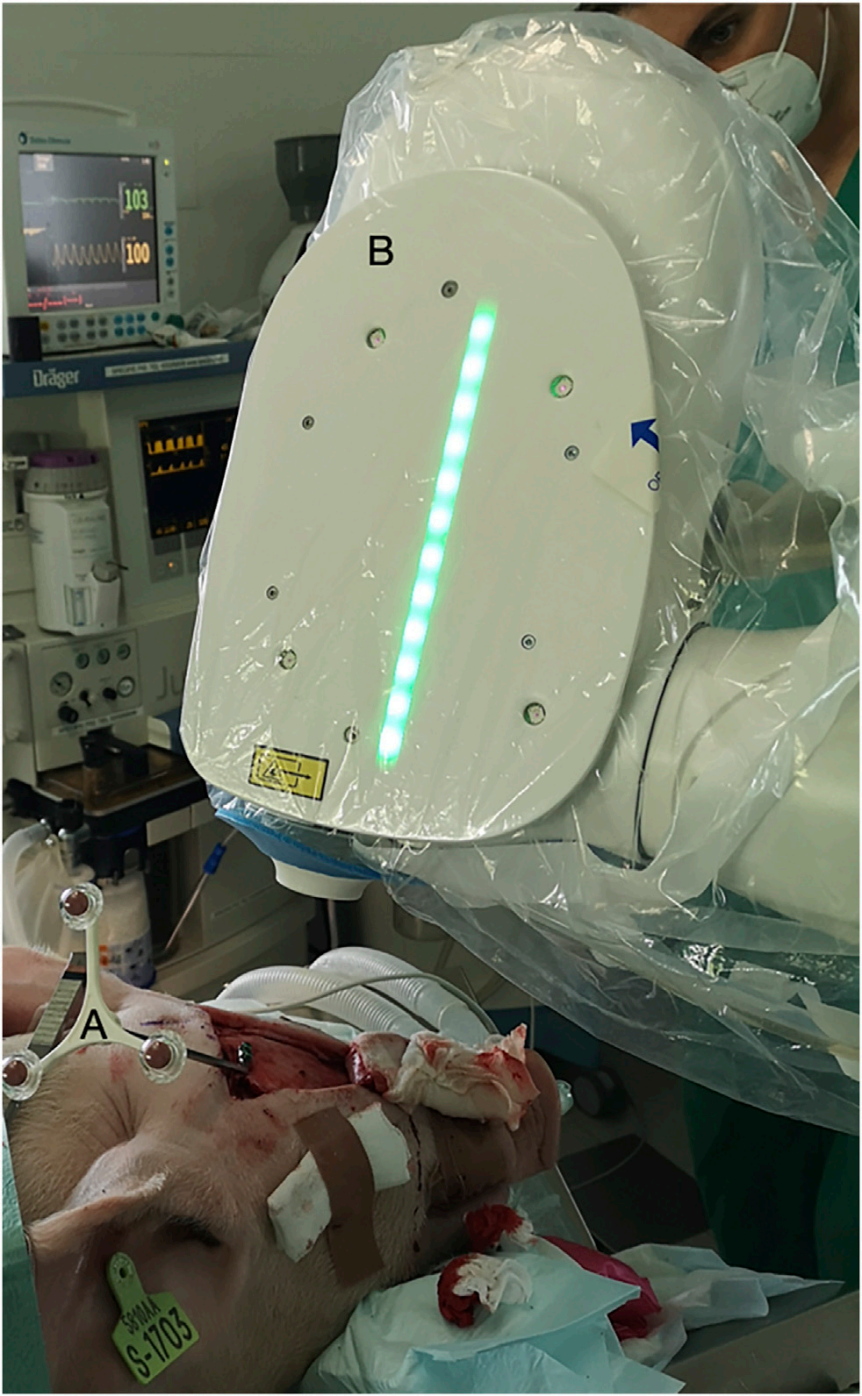
Sterile robotic laser device in action. **(A)** Navigation Marker. **(B)** Sterile CARLO.

The cold ablation robot-guided laser osteotomy (CARLO, AOT, Basel, Switzerland, Figure 3) was performed with a 2.94 µm Erbium:YAG laser with approximately 0.8 mm focal diameter and has a cooling spray integrated to create a fine, sterile sodium chloride film to cool the photo ablated tissue. The laser device integrates optical coherence tomography (OCT) imaging capability, combined with automatic processing, allowing the device to detect the cut’s shape and depth being performed. The OCT laser has a center wavelength of 1.3 µm at a measurement range of 2.2 mm. The full laser system is integrated into one head which is mounted on a KUKA LBR MED robotic arm (KUKA, Augsburg, Germany) which provides lateral repeatability with less than 0.15 mm variation and angular repeatability with less than 7 mrad variation. The robot is guided by a navigation system based on two tracking cameras that observe the fiducial marker attached to the specimen and a fiducial marker integrated into the laser head.

### Surgical Procedure

**FIGURE 4:**
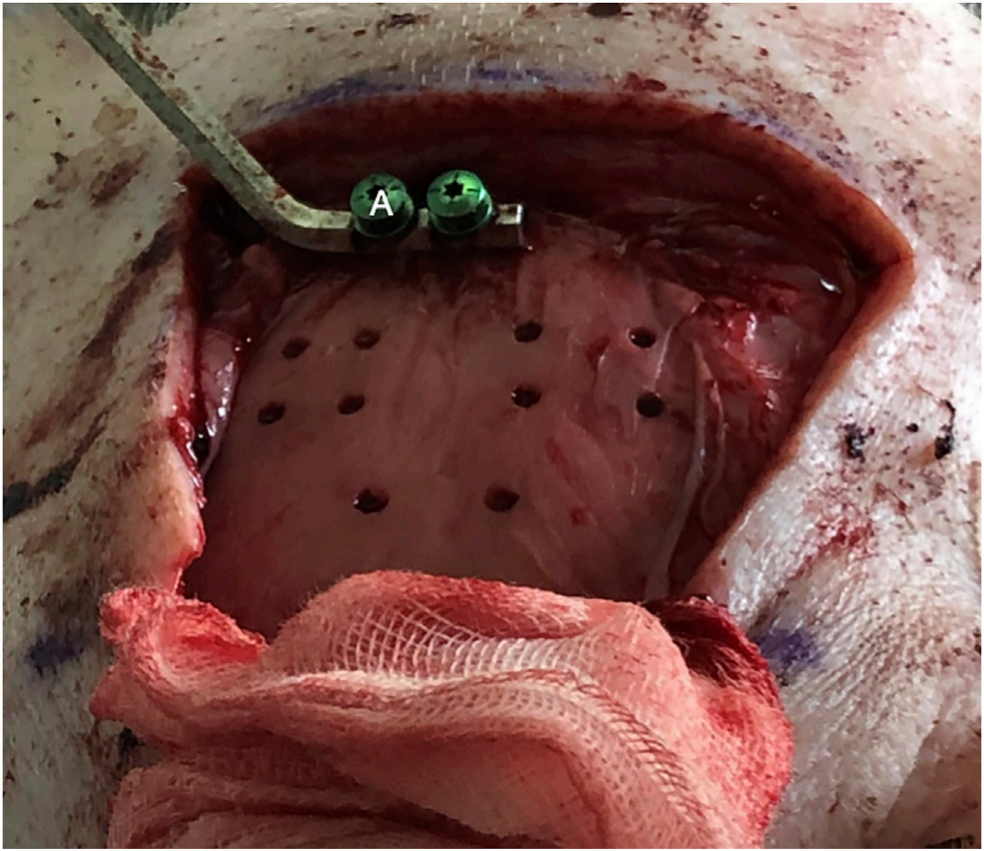
Bone channels in specimen. The two below are set with angulation. **(A)** Navigation marker.

**FIGURE 5:**
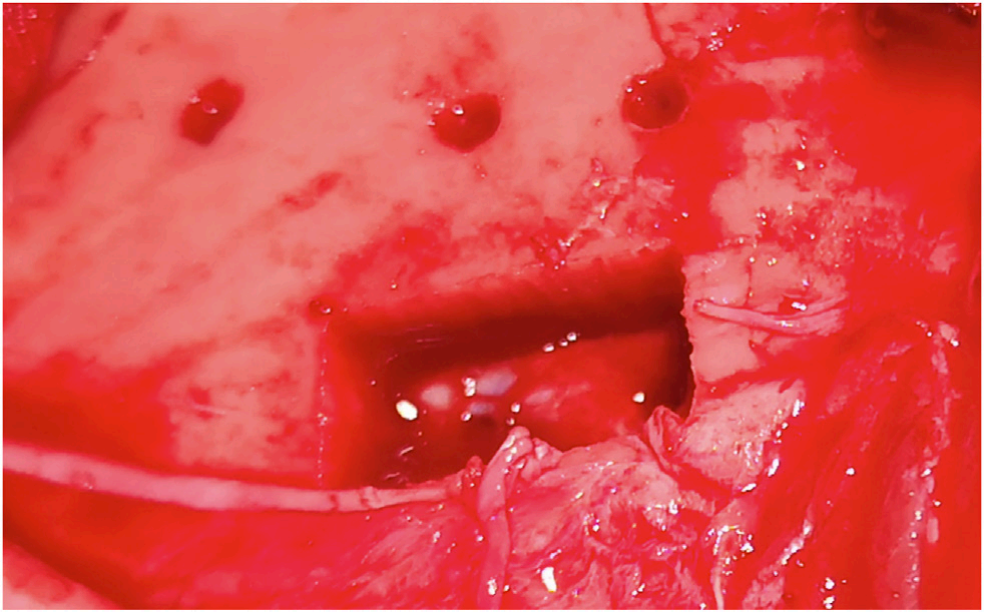
Ad-hoc planned rectangle-shaped craniotomy to evaluate dural damage.

The bone channels were cut *via* laser ablation, as it has been previously demonstrated for the application in maxillofacial surgery (Augello et al., 2018a). Multiple holes suitable for stereoelectroencephalography (SEEG) bone channels were performed on each pig (Figure 4). Craniotomies were planned ad-hoc, a circle-shaped and a rectangle-shaped, which cut out SEEG holes to evaluate dura damage (Figure 5). Controlled dura damage was planned ad-hoc and executed along two lines on intact and exposed dura. Two lines were cut with two different laser power settings with varying shots from one to three per line segment. The surgeon evaluated the grade of damages. Damage to the dura was macroscopically assessed by either seeing the side of impact (minor) or perforating the dura (severe). Minor damage to the cortex was reported if the sight of impact was macroscopically visible, and severe damage was reported if perforation occurred. Trajectories through blood vessels were performed at the end of the surgical procedure before terminating the animal.

### Termination of Animals

The termination protocol was performed while the pig was under anesthesia with an intravenous administration of sodium pentobarbital overdose (≥ 80 mg/kg).

## Results

Nineteen SEEG bone channels were cut in both specimens; three of the channels in a 45°angle configuration. All channels were executed according to the planned cutting strategy. Both anesthesia protocols did not show any irregularities during the operation time. SEEG bone channels and craniotomies were generated. The lengths of the cut bone channels were from 4.4 to 8.2 mm.

### Damage to Dura and Cortex

**FIGURE 6:**
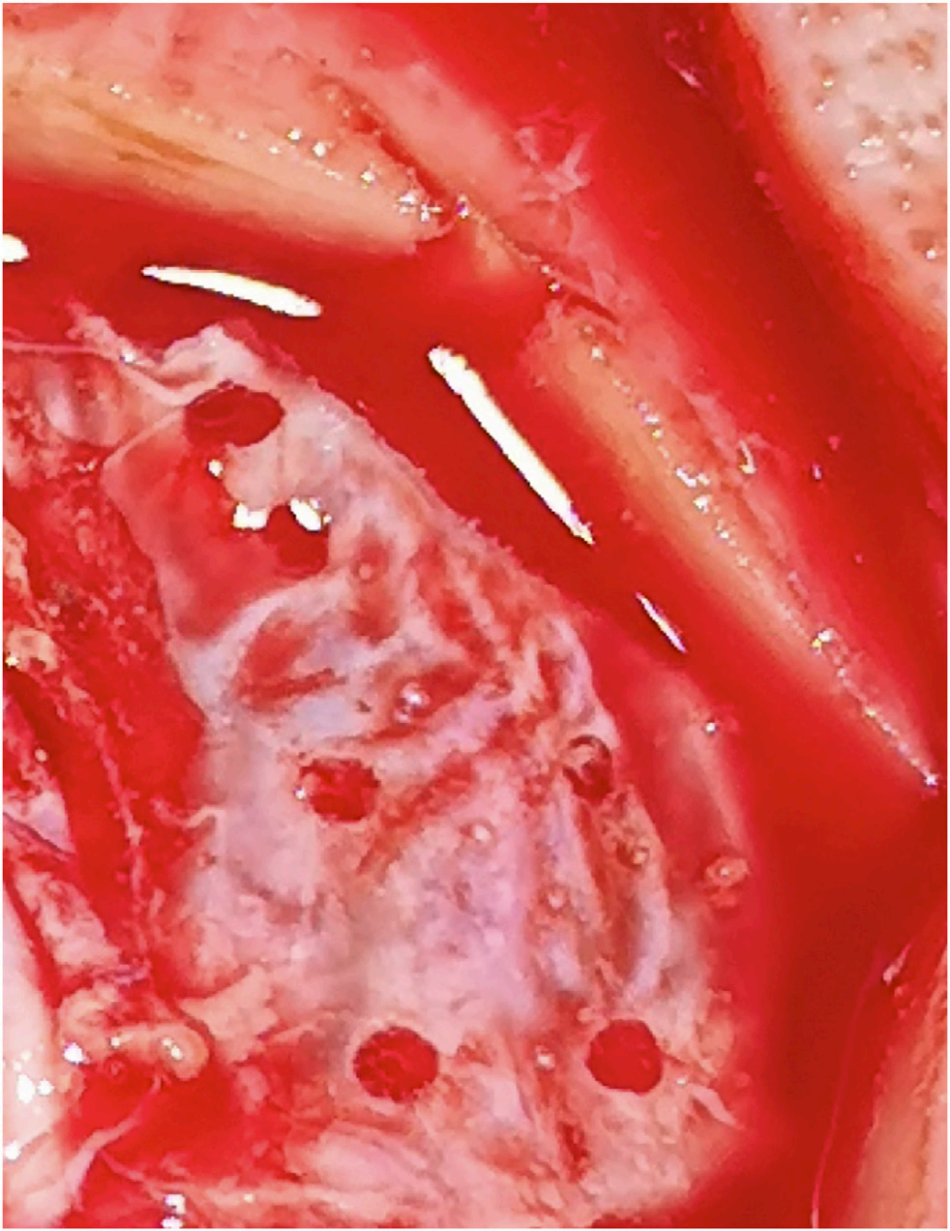
The dura was punctured with one, two, and three laser beams, respectively.

**FIGURE 7:**
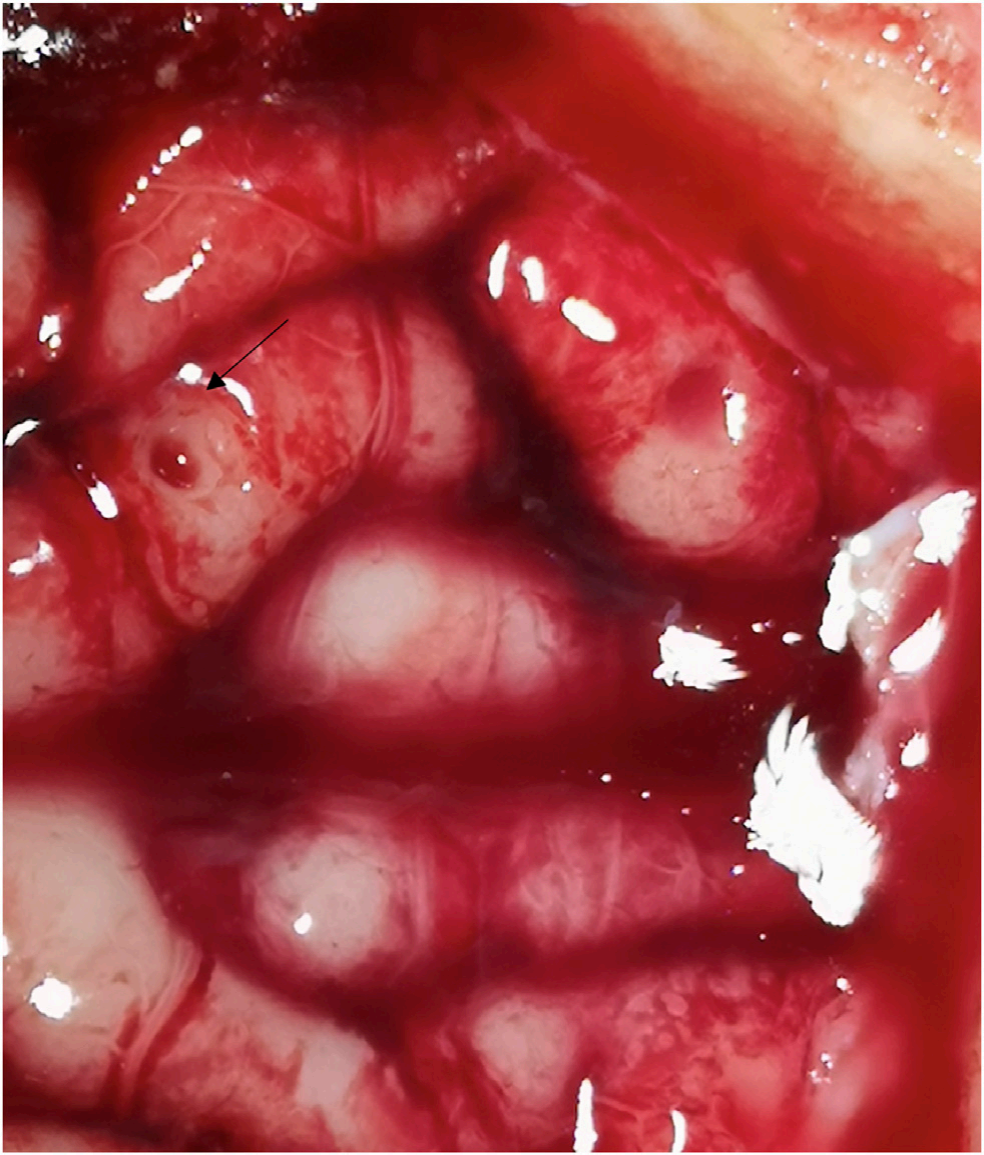
Cortex damage after three laser beams with higher power settings (arrow).

The dura was punctured at a power setting of 2.1 W after one, two, and three laser beams, respectively, (Figure 6). However, damage to the dura did not result in bleeding. Cortex was not damaged after one laser beam, but minor damage occurred after two and severe damage after three laser beams (Figure 7). At 1.1 W power setting, the dura showed minor damage after one laser beam and severe damage after two and three laser beams. However, the cortex was not damaged, neither after one, two, or three laser beams (Table 1).

**TABLE 1:** Visual assessment of dura puncturing and cortex damage: severity 0–2.

Cutting laser power (W)	Number of shots	Grade of dura puncturing	Grade of cortex damage
2.1	1	2	0
	2	2	1
	3	2	2
1.1	1	1	0
	2	2	0
	3	2	0

Dura: 0 = no puncturing, 1 = partial pruncturing, 2 = full pruncturing; Cortex: 0 = no damage, 1 = acceptable damage 2 = unacceptable damage.

### Cutting of Bone Channels

In cut-through mode, it was possible to obtain cut through with the reduced power of 1.1 W. However, it was observed that as soon as cut-through was obtained at one spot, moderate cerebrospinal fluid leakage negatively impacted the cutting efficiency and disturbed the OCT signals. Therefore, to cut through the entire lamella, the laser power had to be raised to evaporate the additional cerebrospinal fluid in the bone channel. We tested higher powers of 1.6 and 2.1 W, where the first was determined to balance the lowest possible power for cut through on one side and cutting efficiency on the other side. The co-axial camera showed a live video feed for the CTR. In 16 bone channels, CTR was detected; in three bone channels, it was not. Additionally, screws and depth electrodes were implanted solely guided by the trajectory given by the laser craniotomies. However, the electrode placement could not be validated.

In total, two craniotomies were performed to the bone channels according to the planned cutting strategy, one in each specimen. One was circular and one rectangular. Similar to bone channels, cerebrospinal fluid, and blood inflow occurred with craniotomies. Cerebrospinal fluid influenced cutting efficiency, and the power was raised to 2.6 and 2.1 W.

## Discussion

The benefits of laser osteotomies are high precision and freedom of geometry. However, available systems are not flexible enough for the operating room and the desired parameters involving the freedom of geometry cannot be achieved (Augello et al., 2018a). In recent years, studies tried to combine the laser device with a navigation system in a space-efficient way to make it desirable in the operating room. A navigation system is crucial to achieving high accuracy with the bone channels. The tested device using Er:YAG laser in this study has been successfully investigated in oral and maxillofacial surgery as the first autonomous, contact-free osteotomy device (Baek et al., 2015; Augello et al., 2018b). This device was already tested in an *ex vivo* study for craniotomies (Roessler et al., 2020).

This study used Er:YAG laser which ablates bone while heat to the surrounding tissues is minimal (Berg et al., 2019). In a clinical setting, it revealed a remarkable cutting efficiency without any damage of adjacent soft tissue structures (Stübinger et al., 2009). However, a drawback of laser osteotomies is prolonged operating times due to relatively low cutting speed. In this series of two animals, the first-ever *in vivo* trial for laser cranial osteotomies, the operating time was significantly shorter in the second specimen, allowing various steps to be performed safer and faster.

**FIGURE 8:**
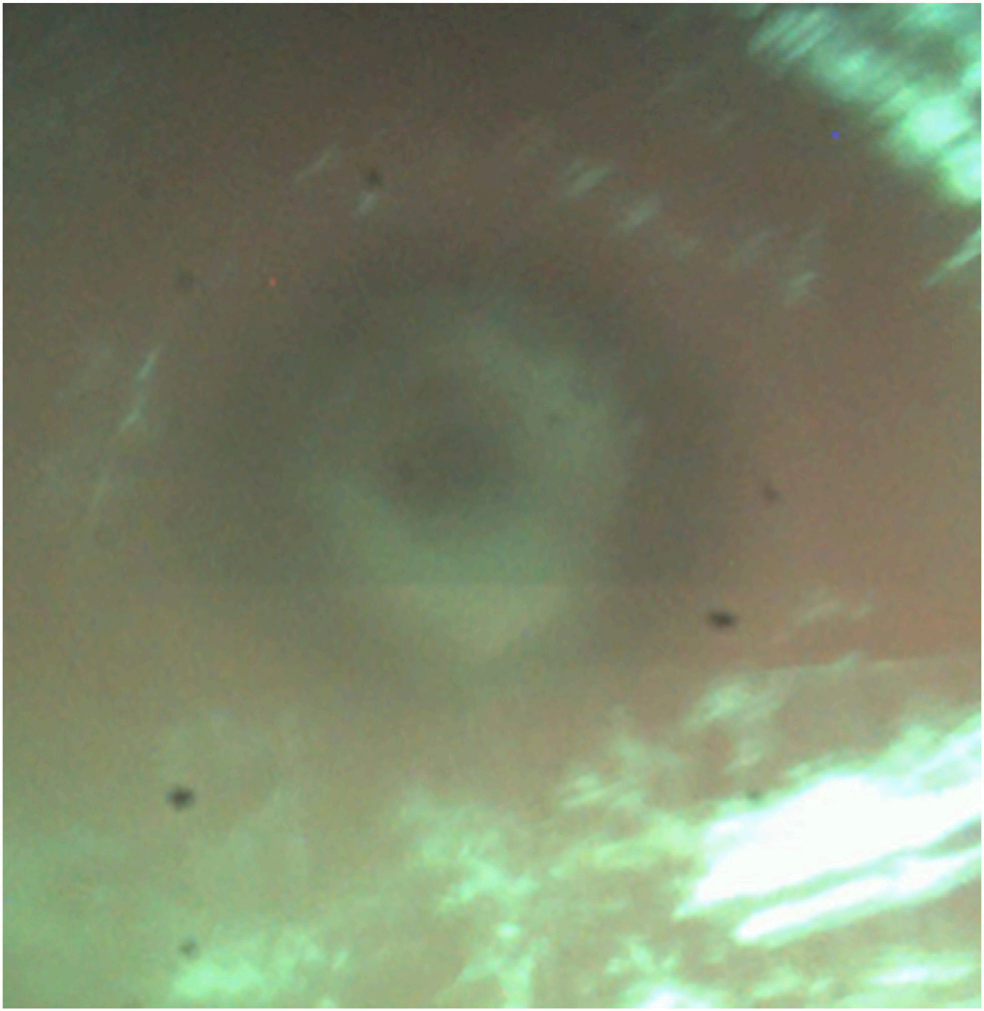
Contrast difference in the middle of a bone channel at cut through.

Recent studies that perform osteotomies with Er.YAG lasers observed difficulties with depth control as visual inspection is limited (Stübinger et al., 2009; Stübinger et al., 2010). Air bone transition signals recorded with OCT in this study were comparable to the *ex vivo* model with the same device, meaning that it is possible to detect cutting depth in real time and operate in remaining bone thickness mode (Roessler et al., 2020). This excludes the erroneous detection of inflowing liquid surfaces originating either from cerebrospinal fluid or blood during the procedure. Inflow from the top occurs due to the cooling spray from the device. The inflow of liquids after the perforation of dura distorted the OCT image. However, CTR might be observed in coaxial video signals typically as contrast differences (Figure 8). The constant surface cooling achieved by the cooling spray integrated into the device is necessary to protect surrounding tissue from carbonizing effects and thermal damage. The inflow and related disturbance of the OCT signals strongly depend on possible pauses’ length during cutting action. There was no difference between bone channels cut with a 90 configuration and the channels with a 45 configuration from a cutting strategy perspective in terms of use and implementation of the laser.

Interactions between the surgeon and the laser device were possible, and this was aided by the in-room memory function of the robotic arm. With the cold ablation robot-guided laser osteotome entering the surgical field from the side with about 90° direction, the surgeon could operate freely from the front. If the surgeon needed more visualization on the operative field, the arm could be retracted and guided back to the same position. This ensures the cooperation of an external device and the surgeon in the already limited space around the operating table.

### Limitations

The generalizability of the results is limited due to the small sample size. Postoperative outcomes and possible clinical side effects could not be detected as this was a non-recovery study. Also, no postoperative imaging or histological analysis was performed. Therefore, damage to intact physiological tissue was only determined macroscopically by a single observer. However, this study was the first *in vivo* study performing cranial bone channels with a contact-free, cold ablation, robot-guided laser osteotome with ways to visualize depth control.

## Conclusion

The dura was damaged, but no uncontrolled bleeding occurred. The cortex was not damaged. Inflowing liquid disturbed OCT signals, and therefore the current cutting through method could not be reliably detected. Video imaging is a candidate for observing cut through. OCT and video imaging may be used for depth control to implement an updated SEEG bone channel cutting strategy.

## Data Availability

The raw data supporting the conclusion of this article will be made available by the authors, without undue reservation.
